# Very low monomethyl fumarate exposure via human milk: a case report—a contribution from the ConcePTION project

**DOI:** 10.3389/fpubh.2024.1393752

**Published:** 2024-07-02

**Authors:** Martje Van Neste, Nina Nauwelaerts, Michael Ceulemans, Benedikte Cuppers, Pieter Annaert, Anne Smits, Karel Allegaert

**Affiliations:** ^1^Clinical Pharmacology and Pharmacotherapy, Department of Pharmaceutical and Pharmacological Sciences, KU Leuven, Leuven, Belgium; ^2^Child & Youth Institute, KU Leuven, Leuven, Belgium; ^3^Drug Delivery and Disposition, Department of Pharmaceutical and Pharmacological Sciences, KU Leuven, Leuven, Belgium; ^4^Teratology Information Service, Netherlands Pharmacovigilance Centre Lareb, ‘s-Hertogenbosch, Netherlands; ^5^BioNotus GCV, Niel, Belgium; ^6^Department of Development and Regeneration, KU Leuven, Leuven, Belgium; ^7^Neonatal Intensive Care Unit, University Hospitals Leuven, Leuven, Belgium; ^8^Department of Hospital Pharmacy, Erasmus University Medical Center, Rotterdam, Netherlands

**Keywords:** case report, breastfeeding, human milk, lactation, pharmacokinetics, multiple sclerosis, dimethyl fumarate, monomethyl fumarate

## Abstract

**Introduction:**

While breastfeeding is recommended, knowledge regarding medicine transfer to human milk and its safety for nursing infants is limited. Only one paper has previously described dimethyl fumarate (DMF) transfer during breastfeeding in two patients at 5 and 6 months postpartum, respectively. The current case report describes maternal pharmacokinetic data of monomethyl fumarate (MMF), the active metabolite of DMF, and infant exposure estimations of MMF at 3 months postpartum.

**Methods:**

A 32-year-old Caucasian woman started DMF therapy (120 mg, 2x/day) for multiple sclerosis at 3 months postpartum, after weaning her infant from breastfeeding. On day 99 after birth, the patient collected four milk samples over 24 h after 6 days of treatment at the initial dose. Additionally, a single maternal blood sample was collected to calculate the milk-to-plasma (M/P) ratio. The samples were analyzed using liquid chromatography coupled with the mass spectrometry method.

**Results:**

A wide range of measured steady-state concentrations of MMF (5.5-83.5 ng/mL) was observed in human milk samples. Estimated daily infant dosage values for MMF, calculated with 150 and 200 mL/kg/day human milk intake, were 5.76 and 7.68 μg/kg/day, and the relative infant doses were 0.16 and 0.22%. The observed mean M/P ratio was 0.059, similar to the M/P ratio predicted using the empirical Koshimichi model (0.06).

**Discussion:**

Combining this case report with the two previously described cases, the estimated infant exposure is low, albeit with relevant intra- and inter-patient variabilities. Research should further focus on infant exposure and safety.

## 1 Introduction

Dimethyl fumarate (DMF), a methyl ester of fumaric acid, is an oral immunomodulatory compound indicated for relapsing–remitting multiple sclerosis (MS). Patients taking DMF have a decreased rate of disability progression, and their annual relapse rate is reduced by half (20% after DMF vs. 40% after placebo intake). It lowers the levels of T cells, dendritic cells, and B cells and induces a shift toward anti-inflammatory activity through the Nrf2 pathway, among others. DMF (molecular weight: 144.13 g/mol) in the gastro-resistant formulation is mainly absorbed in the small intestines. Esterases quickly hydrolyze DMF to monomethyl fumarate (MMF), which has a peak concentration of 2–2.5 h after administration. The plasma protein binding of MMF varies from 27 to 40% in adults, and DMF is later metabolized through the tricarboxylic acid cycle to carbon dioxide (CO_2_), glucose, fumaric acid, and citric acid. Excretion occurs mainly as CO_2_ through exhalation (60%) and secondary by renal (15.5%) and fecal (0.9%) excretion. MMF has a terminal phase half-life of approximately 1 h, and in most adults, circulating MMF is not present anymore at 24 h (1–4). Although MMF is a small, slightly lipophilic (LogP 0.34) molecule with relatively low protein binding, transfer to the slightly more acidic human milk (pH 7.1–7.2) is expected to be low since weak acids such as MMF (pKa 3.31) will concentrate in the plasma (pH 7.4) due to ion trapping (5–7).

After the first large multinational prospective study ‘Pregnancy in MS (PRIMS)’, it is no longer believed that pregnancy could deteriorate the progression of MS in women. On the contrary, a decrease in relapse rate was observed during pregnancy without pharmacotherapy (8, 9). However, during the first 3–4 months postpartum, an increase in the risk of relapses was reported, while exclusive breastfeeding is considered to play a possible protective role in this relapse risk. This protective factor might be explained by the fact that mothers with higher disease intensity are more likely to opt for formula feeding to restart pharmacotherapy (8).

Despite this higher disease activity after delivery and the protective impact of exclusive breastfeeding, limited data are known on the safety of MS pharmacotherapy during lactation. Interferon beta and glatiramer acetate are considered compatible with breastfeeding, although based on limited evidence. At present, DMF is not recommended during breastfeeding, and discontinuation of breastfeeding is advised (8, 9). Only one article has described the transfer of MMF into human milk. Ciplea et al. described a case series of two women taking DMF during breastfeeding: a 35-year-old woman who restarted oral DMF therapy at 6 months postpartum after weaning her infant from breastfeeding, and a 36-year-old patient who discontinued breastfeeding at 5 months to restart DMF treatment. Both women continued to pump so that human milk samples could be collected on the 8^th^ day of using 240 mg DMF, twice daily. Human milk concentrations of MMF (average concentration: 7.5 and 2.7 ng/mL) and relative infant dose (RID: 0.019 and 0.007%) were defined and seemed to be low (10).

Breastfeeding is associated with multiple health benefits for maternal and infant health (11). Therefore, the World Health Organization’s (WHO)^1^ recommendations state that infants should be exclusively breastfed up to 6 months of age and partially breastfed, combined with complementary foods, until a minimum of 2 years of age. Overall, the use and safety of medicines in breastfeeding mothers and the transfer of medicines to their milk have been largely understudied (12). As a result, medicines are often used off-label in this population, and infants can be exposed to unknown risks (13). Furthermore, mothers sometimes choose to postpone their pharmacotherapy and ignore their own health or to interrupt or quit breastfeeding. This case report is part of the UmbrelLACT study established within the Innovative Medicine Initiative (IMI) ConcePTION,^2^ with the aim of limiting uncertainties regarding the effects and exposure of specific medicines during pregnancy and lactation for which knowledge is currently marginally present (14).

The objective of this case report was to determine the concentration of MMF in human milk at 3 months postpartum, to calculate the milk-to-plasma (M/P) ratio of MMF, and to estimate the exposure to a 3-month-old infant. This article is written following the CARE guidelines and ‘The guidelines for reporting cases of medication use during lactation’ (15, 16).

## 2 Methods

This brief research report describes a patient included in the UmbrelLACT protocol (NCT06042803), for which the methods have been described earlier in more detail (14). Approval of the Ethics Committee Research UZ/KU Leuven (S67204) and biobank has been received (14). The patient signed the written informed consent form.

Study information regarding this clinical lactation study is shared via multiple channels, i.e., University hospitals in Leuven, the Belgian pregnancy registry (BELpREG), or other health facilities. After inclusion, clinical data of the mother, e.g., anthropometry, comorbidities, pregnancy and breastfeeding data, and medicine intake during the 3 days before samples were collected using a self-reporting questionnaire.

### 2.1 Sample collection

The human milk samples were collected over a period of 24 h in a steady state. Sampling occurred at home with an electrical breast pump. For each expression session, the total milk volume was reported, and a sample (±10 mL) was taken for analysis. The milk samples were stored in the fridge at the patient’s home (4°C) for a maximum of 24 h.

A maternal blood sample was taken after collecting the last milk sample, in an Ethylenediaminetetraacetic acid (EDTA) tube.

The samples were transported on ice and were subsequently stored at −80°C. Bioanalysis of the samples was conducted using the liquid chromatography with mass spectrometry (LC–MS/MS) method.

### 2.2 Bioanalysis method for monomethyl fumarate (MMF)

Mass spectrometry measurements were performed using a Dionex UltiMate 3,000 LC System (Thermo Scientific Bremen, Germany) coupled via heated electrospray ionization to a Q Exactive Orbitrap mass spectrometer (Thermo Scientific).

Milk or plasma samples of 150 μL were extracted in 80/20 methanol/water with 13C5-D5-15N Glutamic acid as the internal standard. The same procedure was applied to the spiked blank human milk samples used for the calibration curve. These were left overnight at −80°C and then centrifuged for 10 min at 13000 rpm. A volume of 200 μL of supernatant was added to 800 μL of chloroform, after which the samples were vortexed, left to separate at 4°C for 30 min, then vortexed and left to separate again at 4°C for 30 min. Subsequently, 100 μL of the upper phase was transferred to an MS vial and injected into a 15-cm C-18 column (Acquity UPLC-HSS T3 1. 8 μm; 2.1 × 150 mm, Waters). A step gradient was carried out using solvent A (10 mM TBA and 15 mM acetic acid in Milli-Q) and solvent B (100% methanol). The gradient started with 5% of solvent B and 95% of solvent A and remained at 5% B until 2-min post-injection. A linear gradient to 37% B was carried out until 7 min and increased to 41% until 14 min. Between 14 and 26 min, the gradient increased to 95% B and remained at 95% B for 4 min. At 30 min, the gradient returned to 5% B. The chromatography was stopped at 40 min. The flow was kept constant at 0.25 mL/min, and the column was maintained at 40°C throughout the analysis. The HESI source was operated at negative polarity mode using a spray voltage of 4.8 kV, sheath gas at 40, auxiliary gas at 10, and the latter heated to 260°C. The ion transfer capillary temperature was 300°C. The m/z ratio is 129.0 Da for MMF (at negative ESI ionization) and 157.1 Da for the internal standard. The mass spectrometer operated in full scan (range [70.0000–1050.0000]), and the AGC target was set at 3.0E+006 using a resolution of 140,000. Data collection was performed using the Xcalibur software (Thermo Scientific). The data analyses were performed by integrating the peak areas (El-Maven – Polly – Elucidata).

**Figure 1:**
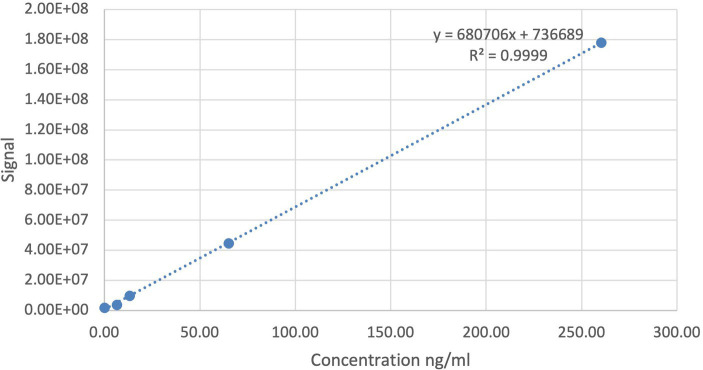
Calibration curve (0–2,000 nM) of the bioanalysis method for monomethyl fumarate (MMF) prepared in blank human milk.

Concentrations were calculated by dividing the abundances, the integrated area of the peak of the compound of interest (MMF), by the slope of the calibration curve (0–2,000 nM) prepared in blank milk (Figure 1). The limit of detection (LOD) was set to correspond to three times the signal in blank human milk without the MMF. The concentration in blank human milk (2.74 ng/mL) was subtracted from the concentrations found in human milk samples and from the LOD, yielding a blank-corrected LOD of 5.48 ng/mL. The blank-corrected limit of quantification (LOQ) was 24.7 ng/mL, which implies that certain sample concentrations might have a larger margin on quantification accuracy.

Additionally, it was observed that DMF added to blank human milk samples produces MMF, which suggests that enzymatic conversion or spontaneous transfer of a methyl by DMF, e.g., to proteins, occurs in human milk, likely reflecting esterase activity in human milk. This esterase activity is hardly relevant for clinical *in vivo* data, as DMF is already metabolized before reaching the systemic circulation.

## 3 Results

### 3.1 Case description: patient information

This case report describes a mother–infant pair, where the infant was weaned before the start of maternal DMF treatment. Therefore, only maternal samples were collected in this case study, and the intake by the infant was estimated. Consequently, there is no observed exposure or safety data in the infant during MMF exposure. The mother is a 32-year-old, primigravida, Caucasian woman who discontinued her treatment since early pregnancy, due to safety concerns during the pregnancy. At the re-initiation of treatment at 3 months postpartum, the patient’s weight was 62 kg and height was 1.87 m, resulting in a body mass index of 17.7 kg/m^2^. While gradually tapering milk production after weaning the infant, samples were collected 6 days after restarting the DMF therapy at the starting dose (2 × 120 mg/day in the first week). The patient had several comorbidities, more specifically Hashimoto’s thyroiditis and thalassemia. Kidney or liver function problems were not reported by the patient. She did not smoke or use recreational drugs, nor did she drink alcohol. The patient followed a pork-free diet.

### 3.2 Timeline

**Figure 2:**
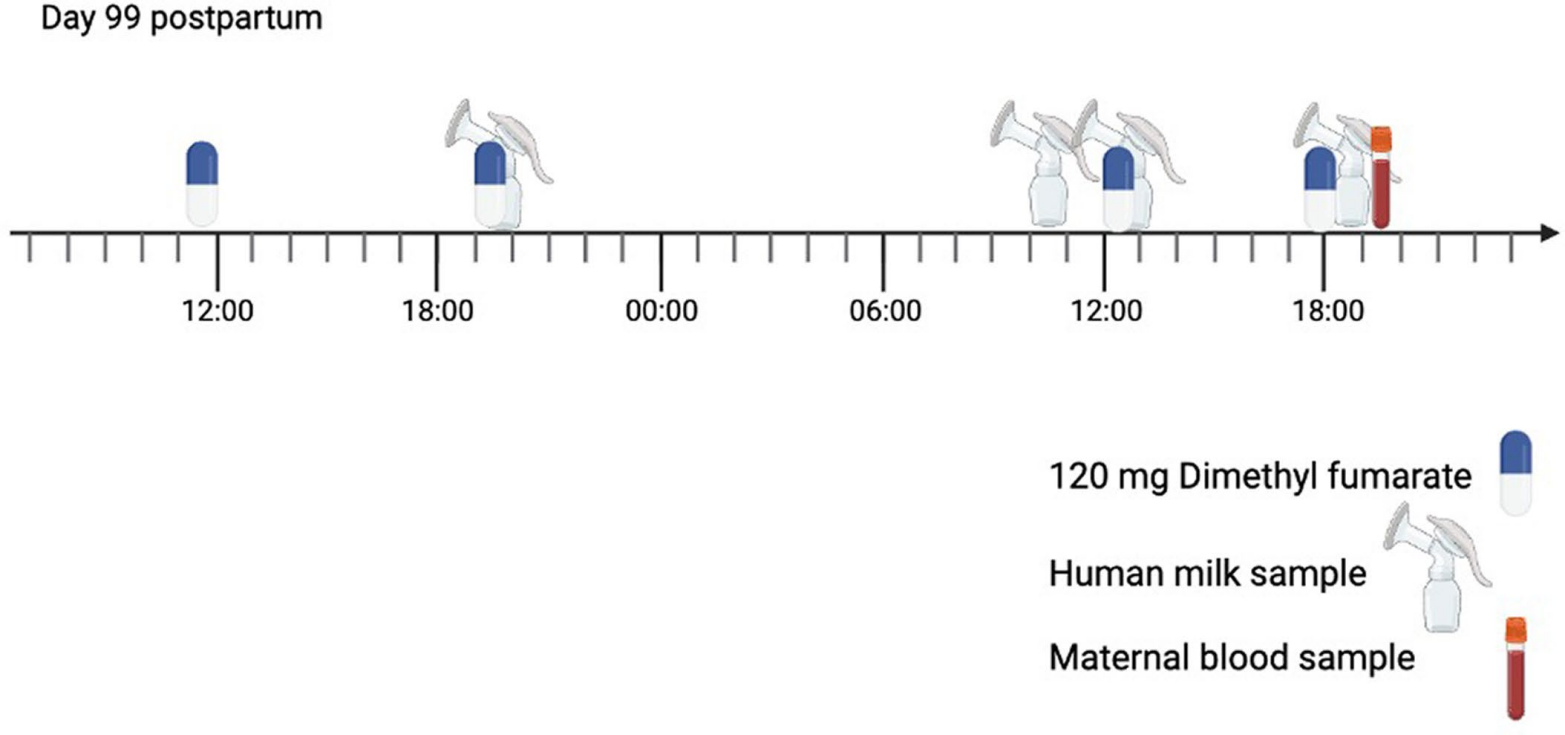
Timeline of the sampling day (start on day 99 postpartum). The mother was taking dimethyl fumarate (DMF) 120 mg twice per day for multiple sclerosis (MS). The mother collected a human milk sample during four expression sessions.

The time points of the sampling day are illustrated in Figure 2.

### 3.3 Therapeutic interventions

The patient used DMF polpharma 120 mg gastro-resistant hard capsules (Polpharma SA Pharmaceutical works, Starogard Gdański, Poland) orally twice daily after a meal. Furthermore, she combined this treatment with Hemafer (357 mg ferric hydroxide polymaltose complex 1x/day, tablets, Fol), Möller’s forte omega-3 (1x/day, capsules), vitamin C (1,000 mg, 1x/d), and vitamin D3 (2000 IU, 1x/d). On day 100 after delivery, the patient did not take vitamin C. These supplements and co-medications are not expected to interact with DMF or MMF.

### 3.4 Maternal data

Clinical data of the mother were collected using a self-reporting questionnaire. The patient reported facial flushing as a common side effect of DMF, which is mentioned in the summary of product characteristics (SmPCs) with an incidence of ≥10% (3).

### 3.5 Samples

Human milk samples were collected over a period of 24 h, starting from the intake of DMF in the evening of day 99 after delivery. These samples were collected at a steady state since the DMF treatment restarted 6 days prior. As the patient’s milk production was decreasing, limited total milk volumes and number of samples were expressed from both breasts during the sampling day.

A maternal blood sample was taken 75 min after collecting the last milk sample. A summary of the human milk sample characteristics is described in Table 1.

**Table 1:** Overview of the timing and the type of the collected samples along with the measured milk concentrations of monomethyl fumarate (MMF).

Days postpartum	Time of sampling (hh:mm)	Time between sampling and last medicine intake (min)	Sample type	MMF concentration (ng/mL)
Day 99 Day 100	19:20 10:30 12:45 18:20 19:35	5 915 15 20 95	Milk Milk Milk Milk Plasma	83.5 <5.5 53.0 11.7 651.3

### 3.6 Human milk pharmacokinetics

**Figure 3:**
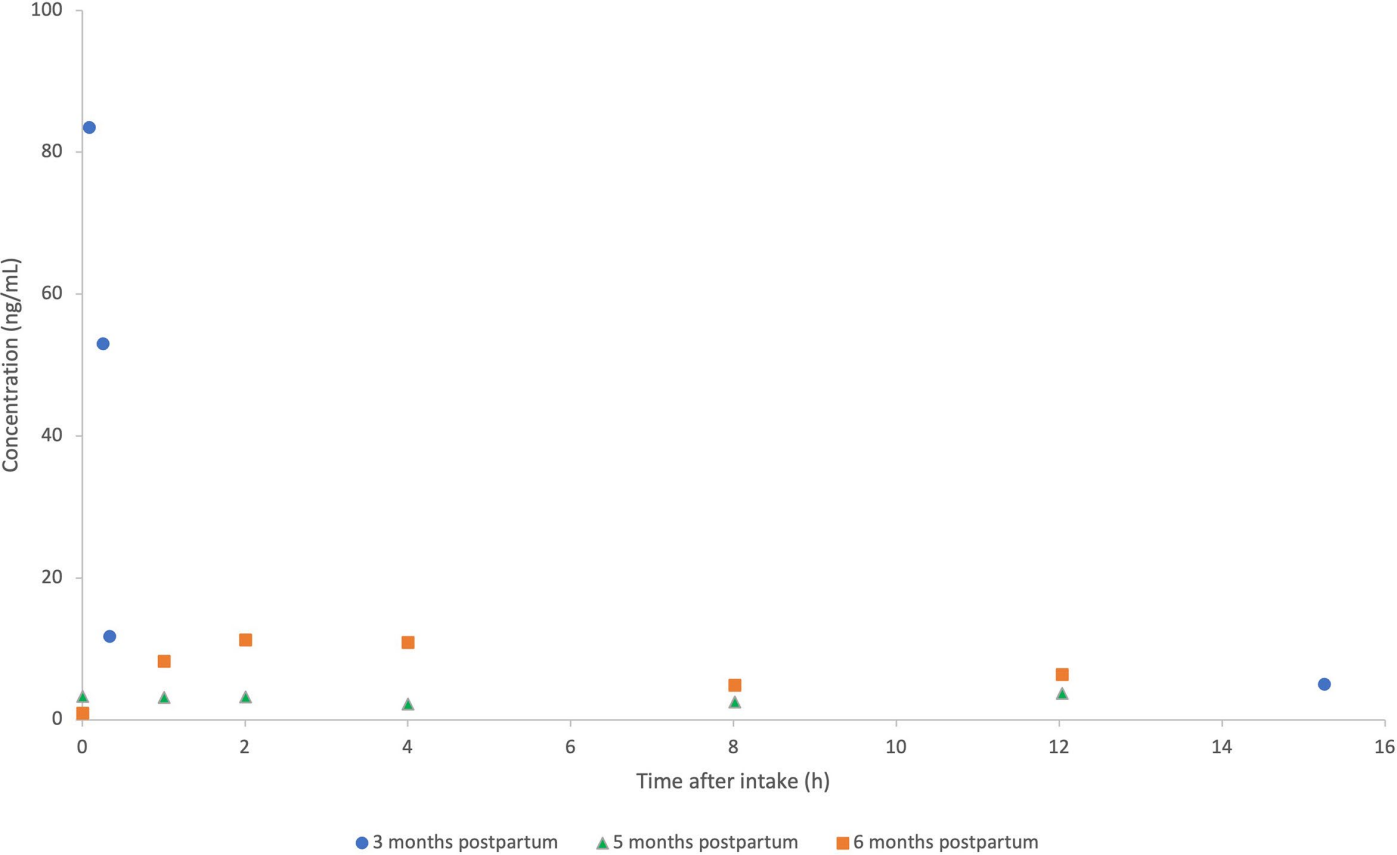
Pooled concentration-time profiles of monomethyl fumarate (MMF) in human milk at 3 months (2×120 mg/day; current case report), compared to concentrations of MMF at 5–6 months postpartum (2×240 mg/day in previously published case series) (10). MMF concentrations (ng/mL) of the patient at 3 months (current case report) (•), vs. 5 months (Δ) and 6 months (⎕) postpartum (case series as previously published by Ciplea et al.).

A total of 4 human milk samples were collected by the patient. The concentration of MMF in the milk samples ranged from 83.5 ng/mL to a concentration under the detection limit of 5.5 ng/mL 2 h prior to the next dose. The concentrations found in our case report are presented in Figure 3 and pooled with the concentrations of the case series previously reported at 5 and 6 months postpartum (data extracted with Webplotdigitizer^3^) (10). All samples in these three pooled cases were collected at steady state, albeit with different daily doses (2×120 mg/day in the current case report and 2×240 mg/day as described by Ciplea et al., respectively).

The concentration of MMF in the blood sample, 95 min after the last DMF intake, was 651.3 ng/mL.

### 3.7 Estimation of infant exposure

The daily infant dose (DID) for a breastfeeding infant after intake of DMF by the mother was calculated using the standardized milk intake of 150 mL/kg/day and milk intake of 200 mL/kg/day to estimate the infant dose in early infancy (Eq. 1).

(1) $$\begin{array}{c} \mathrm{DID}\left(\frac{\frac{\mathrm{\mu g}}{\mathrm{kg}}}{\mathrm{day}}\right)=\mathrm{Average}\;\mathrm{Steady}-\mathrm{state}\;\mathrm{Milk}\;\mathrm{Concentration}\left(\frac{\mathrm{\mu g}}{L}\right)\\ {}*\mathrm{Infant}\;\mathrm{Milk}\;\mathrm{Intake}\left(\frac{\frac{L}{\mathrm{kg}}}{\mathrm{day}}\right) \end{array}$$

In this case, the estimated DIDs of MMF were 5.76 and 7.68 μg/kg/day for a milk intake of 150 mL/kg/day and 200 mL/kg/day, respectively. To calculate the DID, the average steady-state milk concentration in this case report was based on the mean of the four milk samples collected by the patient, as we could not determine an area under the curve (AUC) due to the limited time points.

Calculating the RID was executed by comparing the DID of MMF through human milk, converted to the DID of DMF, and the DMF daily maternal dosage, using 62 kg as the maternal weight (Eq. 2).

(2) $$\mathrm{RID}\;\left(\%\right)=\frac{\mathrm{DID}\;\left(\frac{\frac{\mathrm{\mu g}}{\mathrm{kg}}}{\mathrm{day}}\right)}{\mathrm{Daily}\;\mathrm{Maternal}\;\mathrm{Dose}\;\left(\frac{\mathrm{\mu g}}{\mathrm{day}}\right)/\mathrm{Maternal}\;\mathrm{Weight}\;\left(\mathrm{kg}\right)}*100$$

The RID for DMF based on the DID with a standardized milk intake was 0.16%, and the RID calculating the intake in early infancy (200 mL/kg/day) was 0.22%.

As the efficacy and safety of DMF have not been established in pediatric patients under 10 years of age, the relative infant therapeutic dose (RID_therapeutic_) to compare the DID through human milk to the daily dosages for therapeutic goals, could not be calculated.

### 3.8 Milk-to-plasma (M/P) ratio

We compared the MMF concentration of all four milk samples to the concentration in the maternal plasma sample. These M/P ratios, ranging from 0.01 to 0.13, had a mean of 0.059. In addition, the predicted M/P ratio based on the empirical method developed by Koshimichi et al. was 0.06 (17). This empirical model predicts the M/P ratio based on the physicochemical parameters of the compound of interest, such as lipophilicity (LogP 0.34 and LogD 7.4 −3.09) and molecular weight (130.1 g/mol) (17, 18). These parameters for MMF were extracted from PubChem^4^ and via Chemaxon MarvinSketch (version Europium 7). However, this prediction is less reliable for medicines with a short half-life (5).

## 4 Discussion

This case report presents the concentrations of MMF as observed in human milk, the calculated M/P ratio, and the estimated child exposure to MMF at 3 months postpartum. This case report hereby adds to the very limited literature currently available, as only two cases of MMF transfer to human milk have been previously reported at 5 and 6 months after delivery (10). Furthermore, this is the first time the M/P ratio of MMF was calculated based on observed data and compared to the predicted M/P ratio according to the empirical method by Koshimichi et al. (17).

When comparing the human milk concentrations in our study, we noticed high intra-individual variability during the first hour after intake at a steady state (Figure 3). Notably, high inter-individual variability was also seen between both patients in the previous case series of Ciplea et al., with an AUC of 90 and 33 ng*h/mL, respectively. It is reported that the intake of DMF in combination with food, especially fatty food intake, can delay the peak concentration, but it is reported not to have a relevant effect on DMF subsequent exposure (2, 3). Intake with food is also recommended to reduce flushing, which means that this combination reflects common practices, as in this case.

Even though our patient was taking half the daily dose of the previously published case series, the concentrations found in the human milk of this case are considerably higher, with the highest measured concentration of 83.5 ng/mL (5 min after intake) compared to a C_max_ of 11.2 and 3.7 ng/mL (2 h after intake) in Ciplea et al. This trend is also observed in the DID and RID. In this case report, the calculated DID for the standardized human milk intake (150 mL/kg/day) is 6- to 16-fold higher than the DIDs found in the previous case series. The RID, calculated with a human milk intake of 150 mL/kg/day, is 10-fold higher in our patient (10). In addition, the plasma levels in our patient were high compared to previous observations in patients using 240 mg of DMF twice daily (19).

We can only hypothesize on the underlying causes of these differences and the inter-individual variability. This might be explained by the physiological changes during the postpartum period in women. Some changes during postpartum, e.g., endocrine-driven metabolic clearance, are not completely understood and need further research (20). MMF metabolism by esterases in the gastrointestinal tract, tissues, and blood might be altered postpartum. For instance, the effect of rocuronium, a competitor of acetylcholine, is prolonged during the postpartum period, suggesting lower esterase activity. However, this prolongation can also be explained by the increased body weight, as this effect was not seen when doses were calculated by lean body mass (21). Gastric emptying rate could also play a factor in the absorption pattern of this compound (22), although this is not always observed postpartum (23). The peak concentration of MMF is postponed when the intake of DMF is combined with a fatty meal (3). This delayed absorption could contribute to the observed variability. Furthermore, the possibility of decreased elimination of MMF due to a change in the tricarboxylic acid cycle between 3 and 5–6 months postpartum is highly unlikely. Additionally, the risk of variability in DMF exposure due to pharmacogenetics is considered to be low and therefore, not a probable cause of these results (24). The hypothesis regarding the effect of postpartum physiological changes is further supported by the high concentration in plasma at 95 min after DMF intake compared to the literature (19).

Another hypothesis for the higher concentrations in our patient might be the accumulation of MMF in human milk, as this is a slightly lipophilic metabolite (LogP 0.34). Since our patient expressed milk four times during 24 h, compared to 6 times during 12 h in the case series (10), there might be more accumulation of MMF in the lipid fraction of human milk (5). However, the computed fraction unbound in human milk (f_u,m_ 1.00) counters this hypothesis (17).

Despite the higher values in our study, the RIDs calculated with a human milk intake of 150 mL/kg/day and 200 mL/kg/day are 0.16 and 0.22%, respectively, which is far below the arbitrary threshold for acceptable risk for infants of 10% (25). In line with previous reports, these new findings show no new evidence for concern for DMF therapy during lactation. Furthermore, DMF is not expected to be present in human milk as it is rapidly metabolized into its metabolites, such as MMF. Nonetheless, next to the low RIDs, other aspects should be acknowledged regarding the safety of infants, such as oral bioavailability and potential developmental toxicity (10). Therefore, more data should be obtained to adequately advise DMF treatment to breastfeeding mothers, as high inter- and intra-individual variability has been observed and the maturational physiology of infants and its consequences are not yet completely understood. Moreover, the general health of breastfed infants whose mothers take DMF and possible infant side effects, e.g., flushing and gastrointestinal discomfort, should be studied as well (3). Unfortunately, data on infants’ tolerance during lactation-related DMF exposure were lacking both in this case report and the previously published case series (10).

Due to the nature of clinical lactation studies, it is uncertain if the patient managed to follow the instructions perfectly, a limitation related to the pragmatic design of these studies. For example, it is possible that milk was not fully expressed. This would mean that these calculations are based on concentrations found in foremilk. As foremilk has a considerably lesser fat content than hindmilk and MMF is (somewhat) lipophilic, the concentration measured with fully expressed milk might be higher than in this case report. As the patient had weaned her infant from breastfeeding, the DID could not be calculated using the exact intake volume for this infant. Additionally, the limited number of human milk samples did not allow us to calculate the AUC of MMF in human milk, and the C_max_ could not be determined. Moreover, as all three mothers restarted their treatment after weaning their infant from breastfeeding, the effect of MMF in human milk could not be assessed in the infant, which is another knowledge gap that has to be addressed in the future. However, this report describes the earliest case of DMF treatment during lactation and hence, adds pieces to the puzzle to provide relevant information for evidence-based shared-decision making between health care professionals and patients. Due to the observed variability, future studies are warranted to further determine infant exposure to DMF through human milk. The development of PBPK models is recommended for this compound, as only few data are needed to additionally assess the extent of exposure to this compound in nursing infants (18).

In summary, the estimation of infant exposure is low in our case and the two other previously described cases, despite the relevant intra- and inter-individual observed variability. Research is needed to further assess the exposure and safety of the infant.

## Data availability statement

The datasets presented in this article are not readily available because of concerns regarding participant/patient anonymity. Requests to access the datasets should be directed to MVN, martje.vanneste@kuleuven.be.

## Ethics statement

The studies involving humans were approved by Ethics Committee Research UZ/KU Leuven (S67204). The studies were conducted in accordance with the local legislation and institutional requirements. The participants provided their written informed consent to participate in this study. Written informed consent was obtained from the individual(s) for the publication of any potentially identifiable images or data included in this article.

## Author contributions

MVN: Conceptualization, Data curation, Methodology, Project administration, Writing – original draft, Writing – review & editing. NN: Conceptualization, Methodology, Project administration, Writing – review & editing. MC: Conceptualization, Methodology, Project administration, Writing – review & editing. BC: Conceptualization, Data curation, Methodology, Writing – review & editing. PA: Conceptualization, Methodology, Project administration, Writing – review & editing, Supervision. AS: Conceptualization, Methodology, Project administration, Supervision, Writing – review & editing. KA: Conceptualization, Methodology, Project administration, Supervision, Writing – review & editing.

## Footnotes

^1^ https://www.who.int/health-topics/breastfeeding#tab=tab_1

^2^ https://www.imi-conception.eu/

^3^ https://apps.automeris.io/wpd/

^4^ https://pubchem.ncbi.nlm.nih.gov/
